# Supplementary material for: Direct visualization of critical hydrogen atoms in a pyridoxal 5′-phosphate enzyme
Source: Nat Commun. 2017 Oct 16;8:955. doi: 10.1038/s41467-017-01060-y (PMC5643538; doi:10.1038/s41467-017-01060-y)
Supplement: Supplementary file 1 — Supplementary Information [file 41467_2017_1060_MOESM1_ESM.pdf]

Supplementary Figure 1 AAT crystal packing

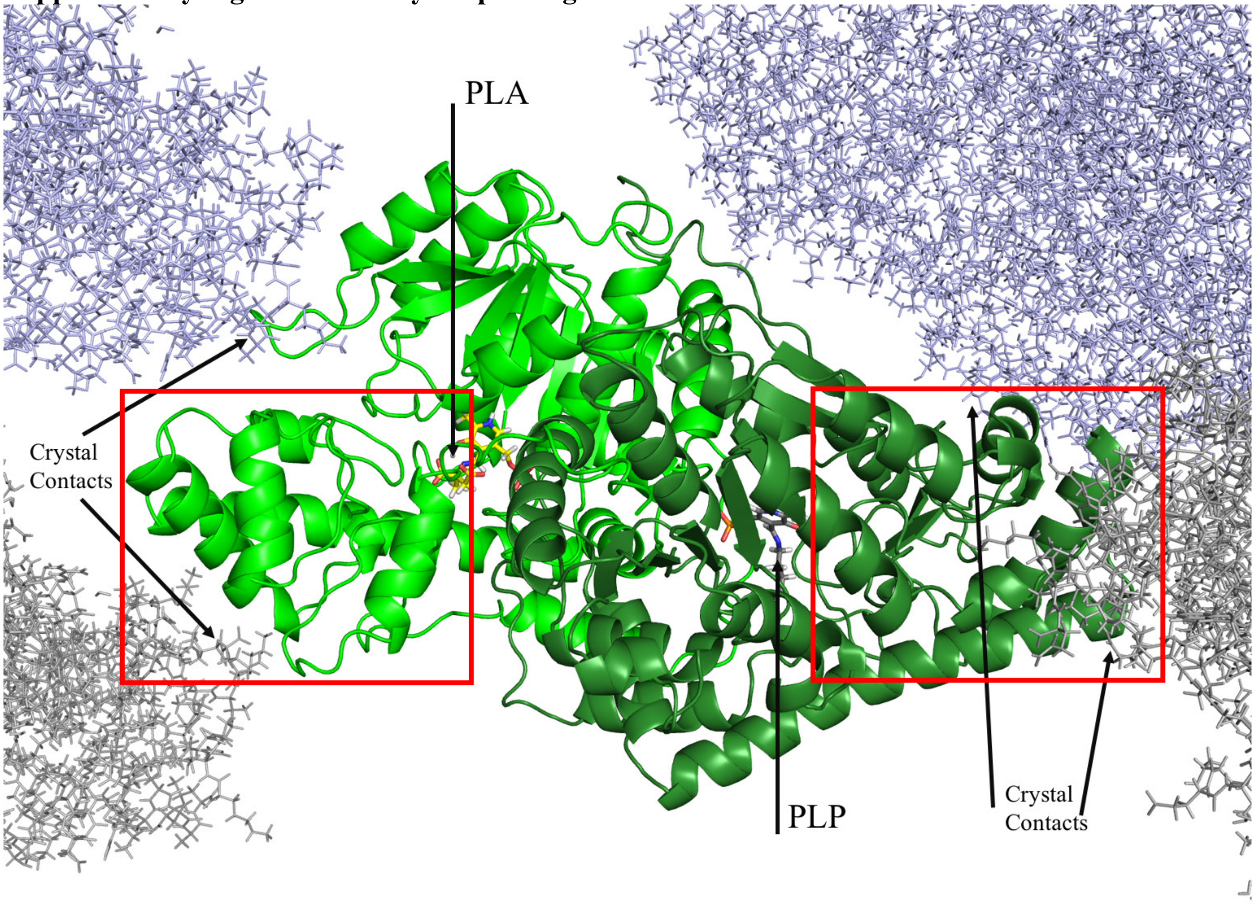

Crystal contacts involving the small domains (highlighted by the red boxes) of each respective monomer; chain A (the external aldimine) in light green and chain B (the internal aldimine) in dark green. The limited contacts in chain A allow for multiple conformations while the extensive contacts in chain B restrain the small domain in the open form.

Supplementary Figure 2 Extended hydrogen bond network near N1-PLP

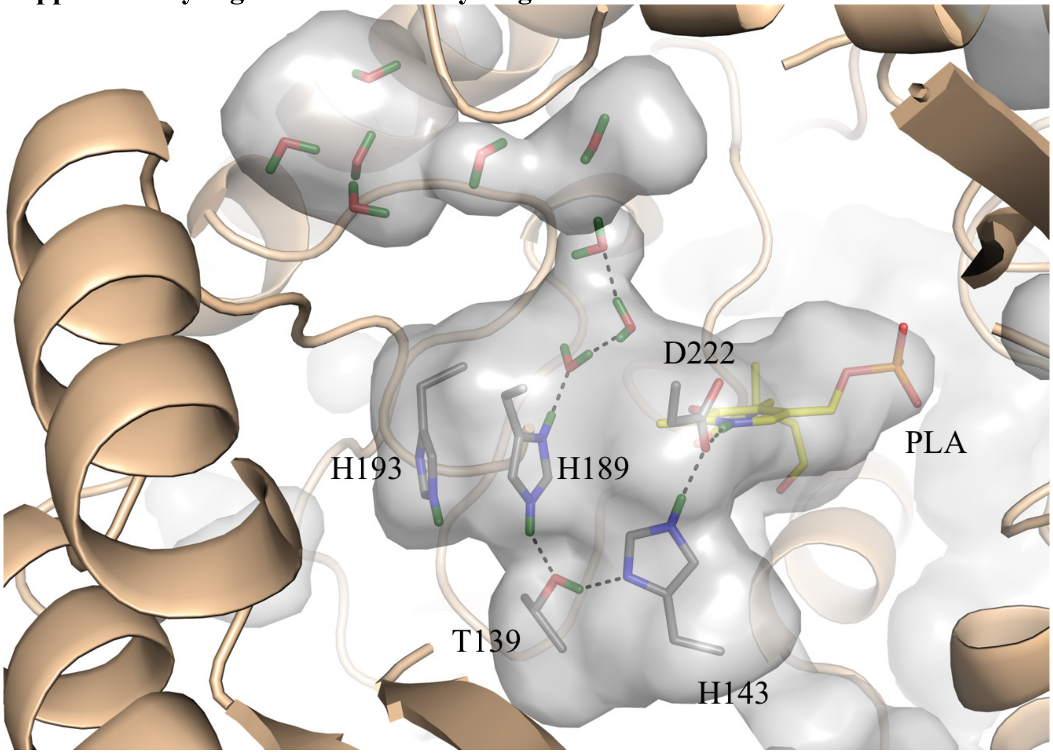

The gray surface represents the void when the atoms shown in sticks are deleted and any additional pre-existing voids in the protein.

Supplementary Figure 3 Unconstrained Optimized geometries of internal aldimine models

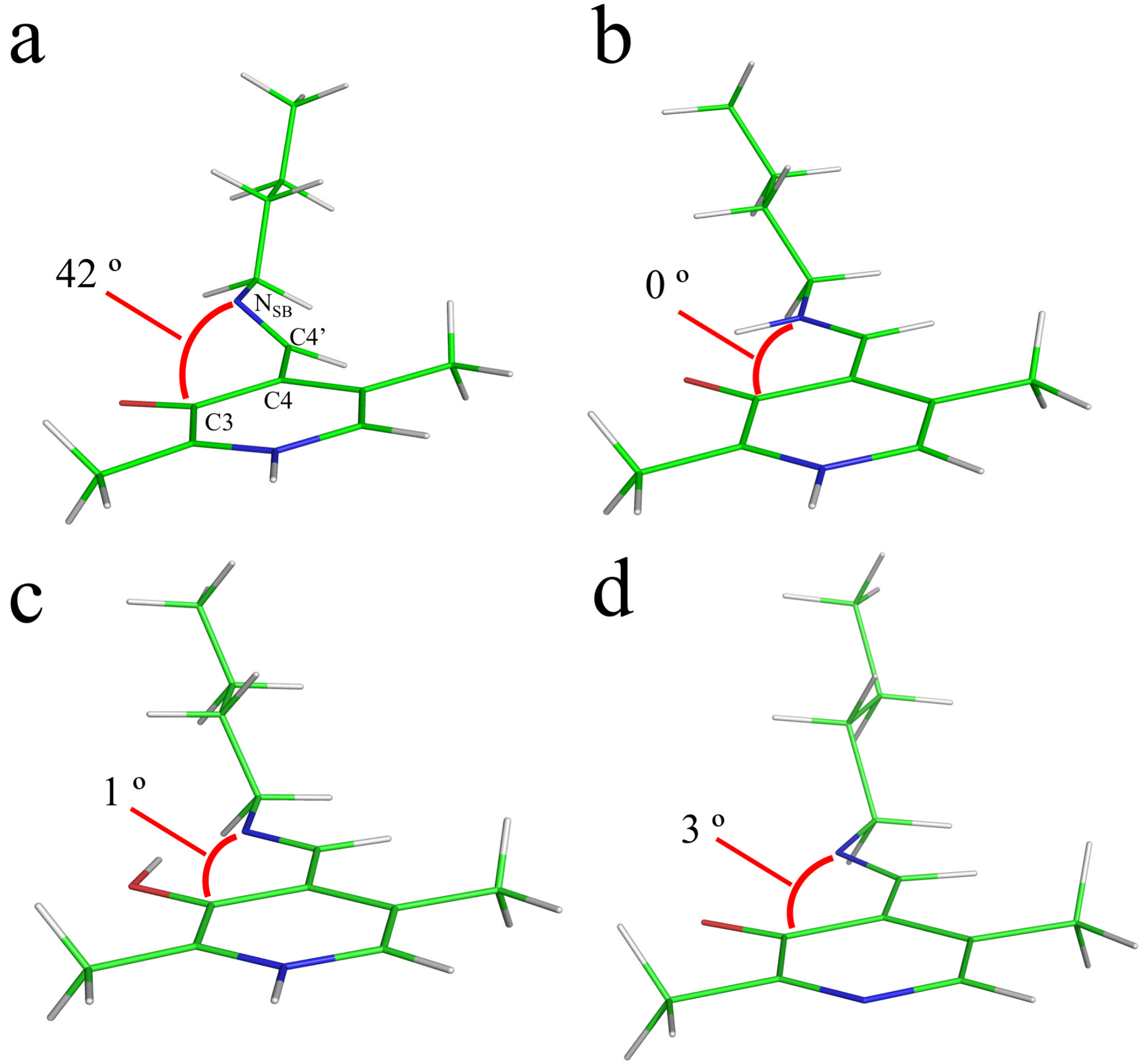

Panel A is the SB deprotonated internal aldimine model, Panel B is the SB protonated internal aldimine model, Panel C is the O3' protonated internal aldimine model, and Panel D is the SB and N1-PLP deprotonated model. In each panel the red arc specifies the C3-C4-C4'-N_{SB} dihedral angle.
