# Supplementary material for: Direct visualization of critical hydrogen atoms in a pyridoxal 5′-phosphate enzyme
Source: Nat Commun. 2017 Oct 16;8:955. doi: 10.1038/s41467-017-01060-y (PMC5643538; doi:10.1038/s41467-017-01060-y)
Supplement: Supplementary file 3 — Description of Additional Supplementary Files [file 41467_2017_1060_MOESM3_ESM.pdf]

Description of Supplementary Files

File name: Supplementary Data 1
Description: XYZ coordinates of the Internal aldimine model with the Schiff base deprotonated

File name: Supplementary Data 2
Description: XYZ coordinates of the Internal aldimine model with the Schiff base and N1 deprotonated

File name: Supplementary Data 3
Description: XYZ coordinates of the Internal aldimine model with the Schiff base protonated

File name: Supplementary Data 4
Description: XYZ coordinates of the Internal aldimine model with the O3’ protonated

File name: Supplementary Data 5
Description: Natural bond orbital analysis of Internal aldimine pH 7.5 model

File name: Supplementary Data 6
Description: Natural bond orbital analysis of Internal aldimine pH 4.0 model
